# Supplementary figures and images for: Time Series Ovarian Transcriptome Analyses of the Porcine Estrous Cycle Reveals Gene Expression Changes during Steroid Metabolism and Corpus Luteum Development
Source: Animals (Basel). 2022 Feb 4;12(3):376. doi: 10.3390/ani12030376 (PMC8833361; doi:10.3390/ani12030376)

Fig. S1

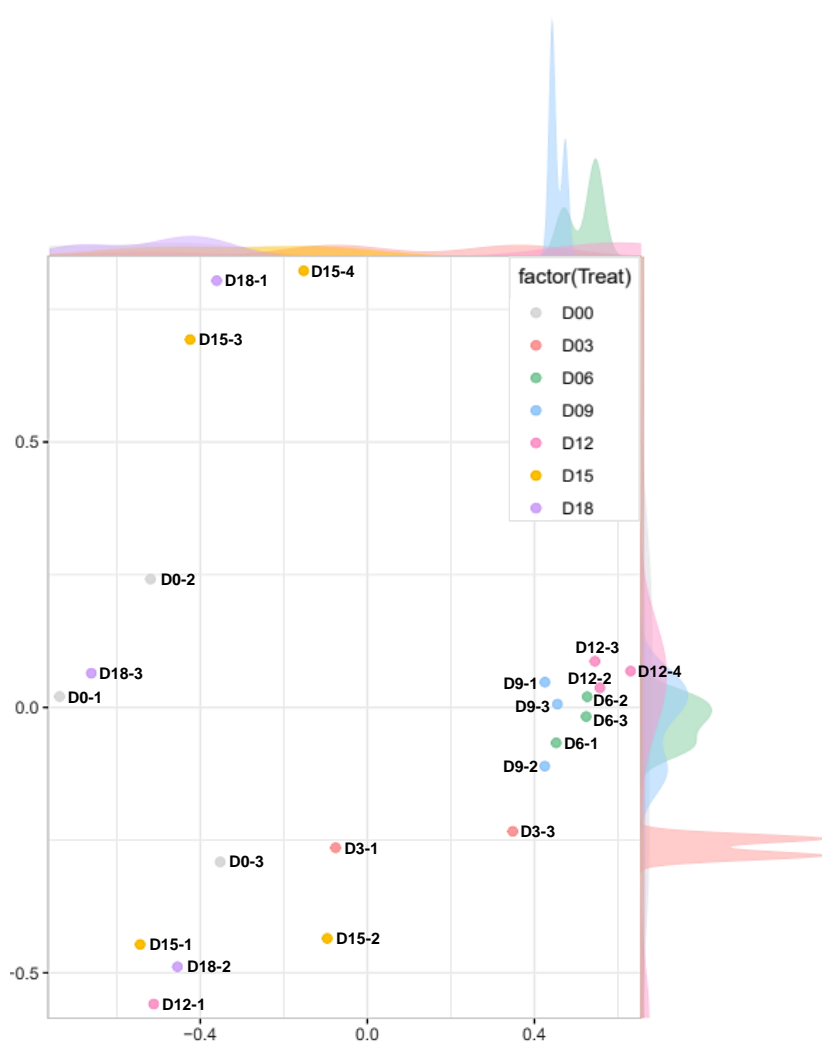

Fig. S2

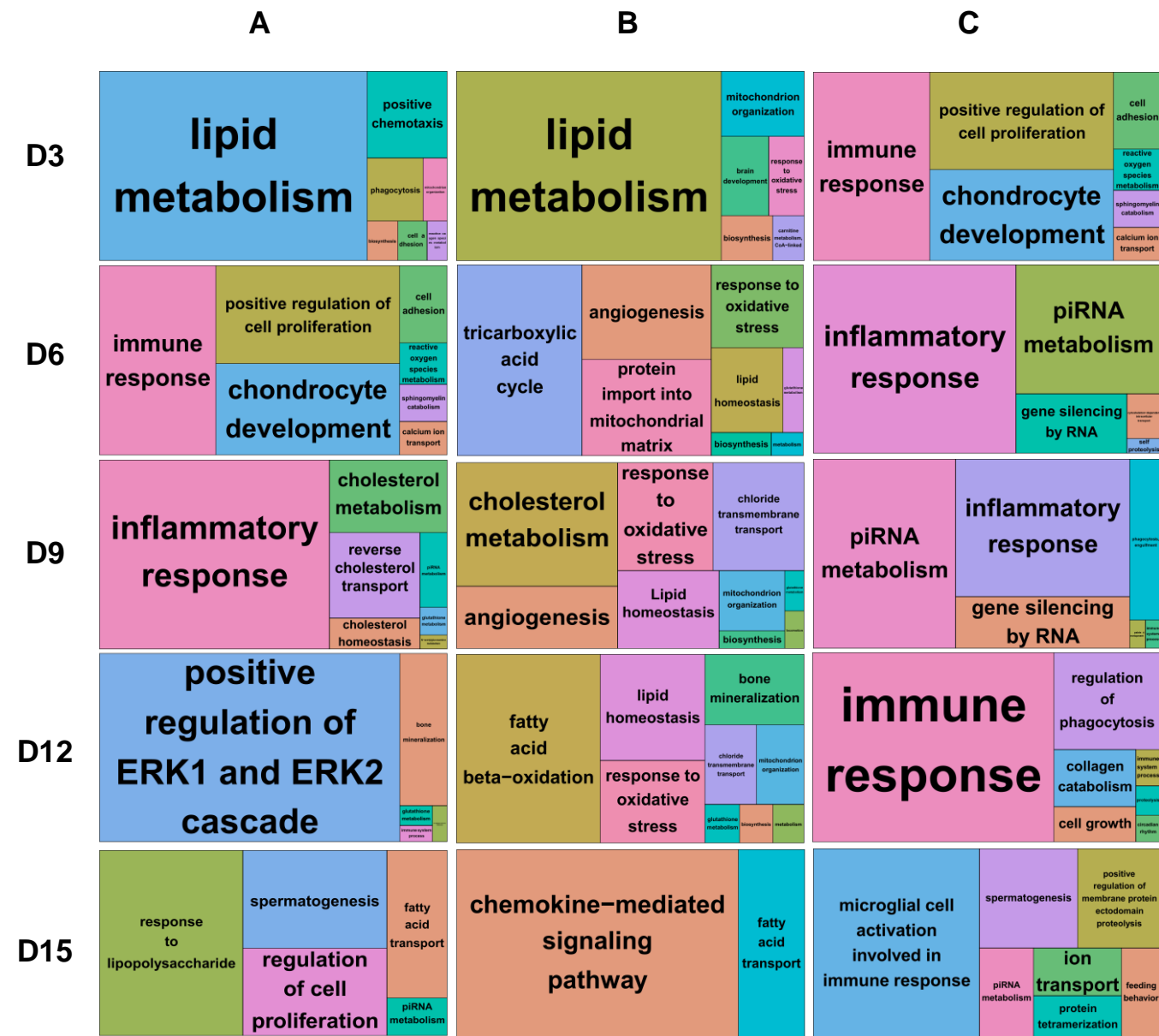

Fig. S3

D3

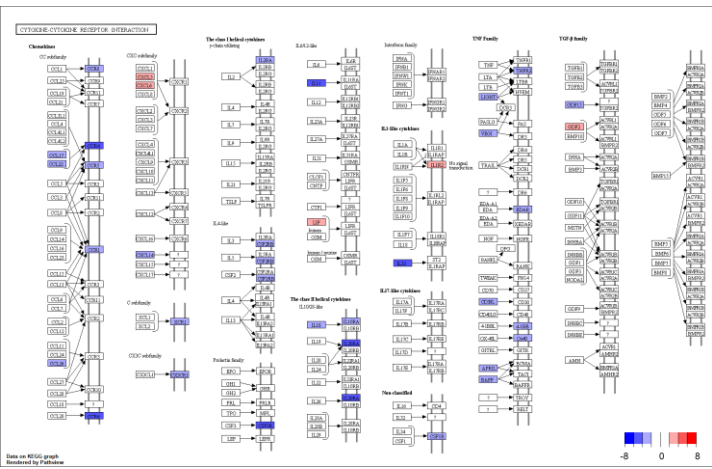

D6

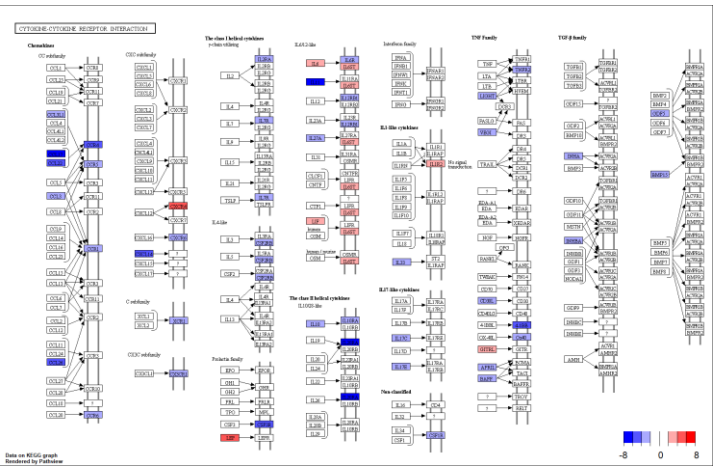

D9

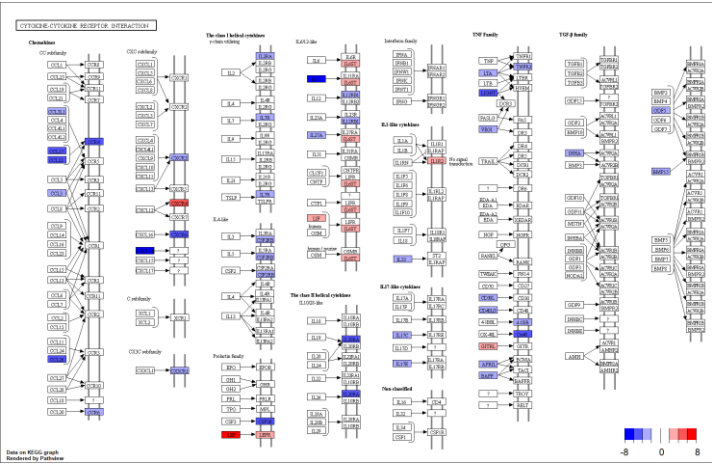

D12

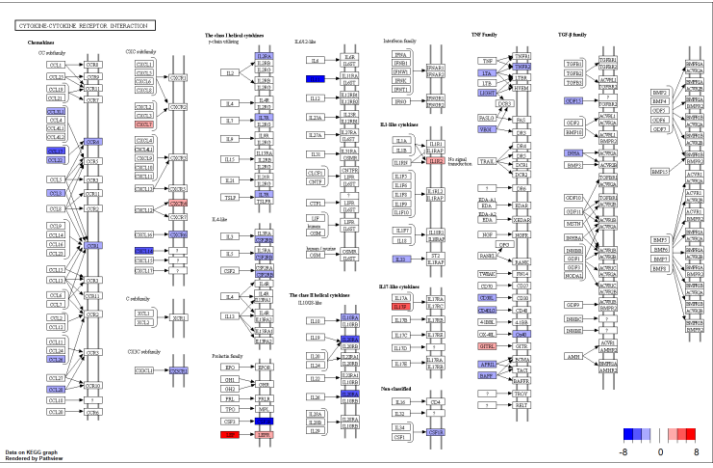

D15

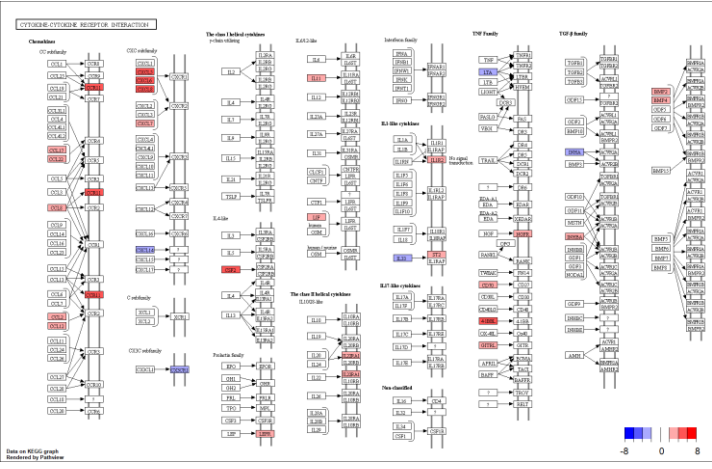

D18

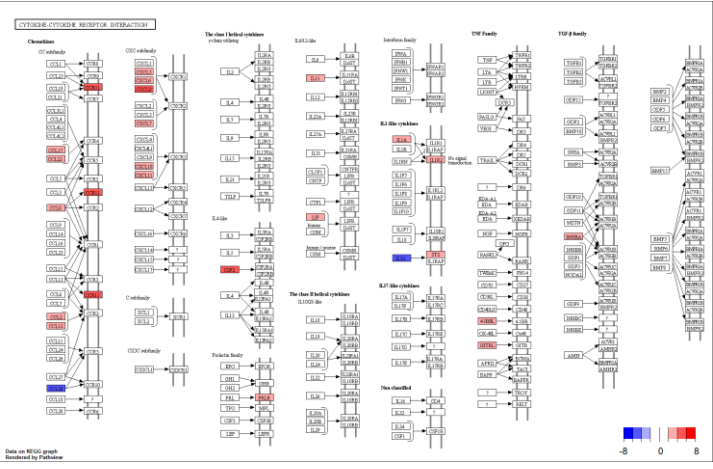

Fig. S4

D3

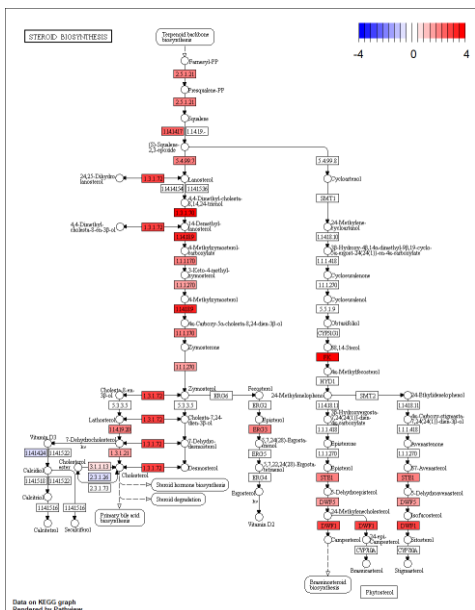

D12

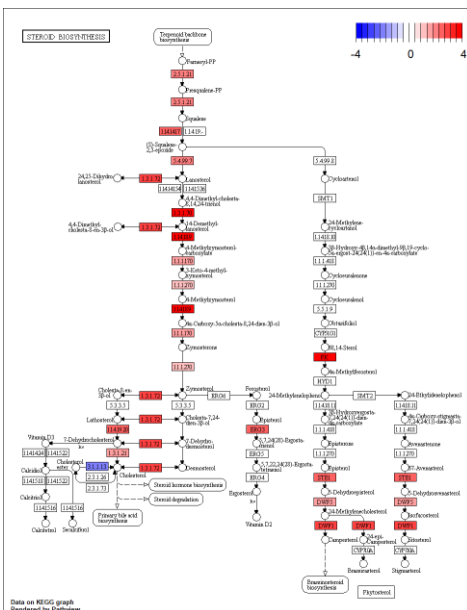

D6

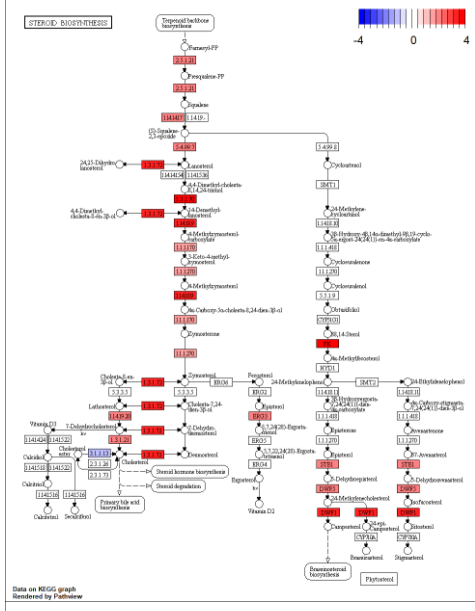

D15

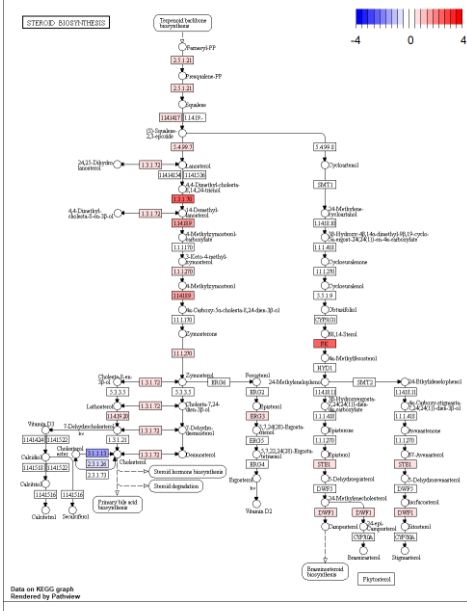

D9

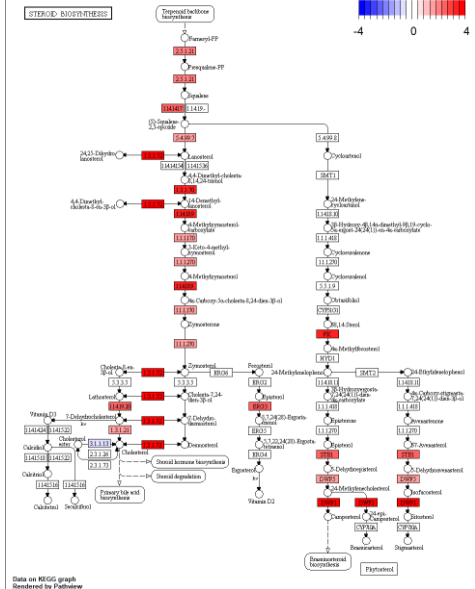

D18

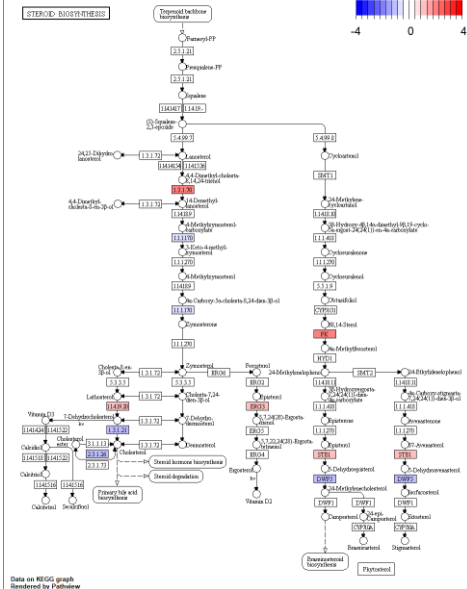

Fig. S5

D3

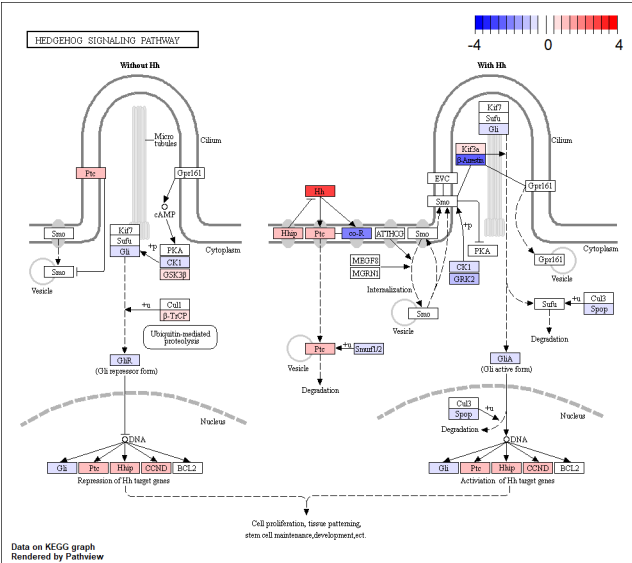

D12

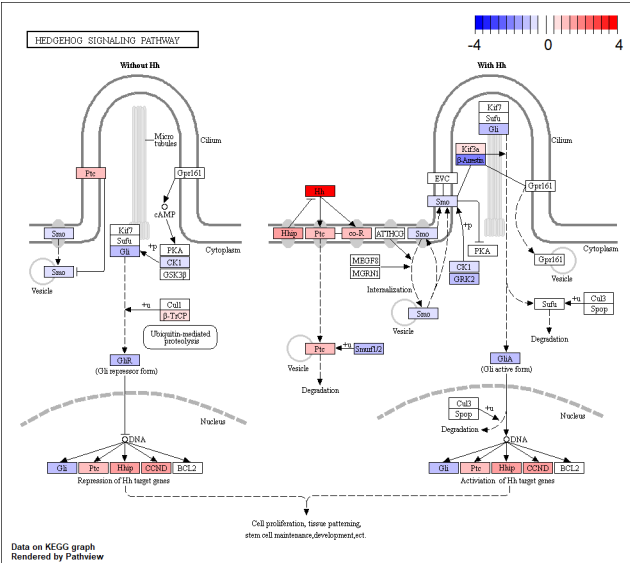

D6

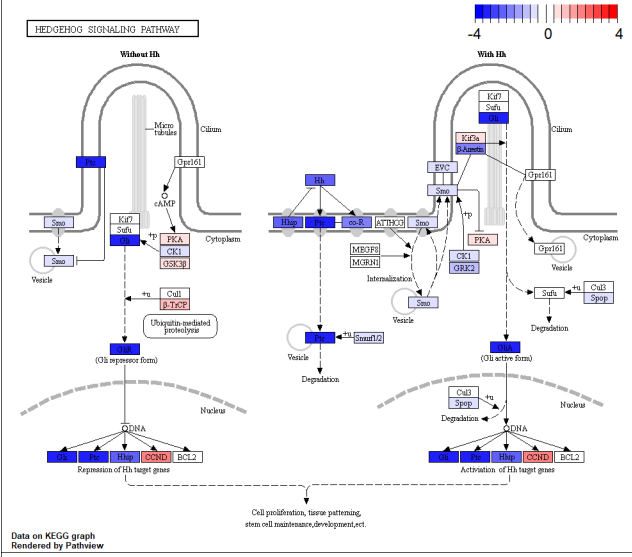

D15

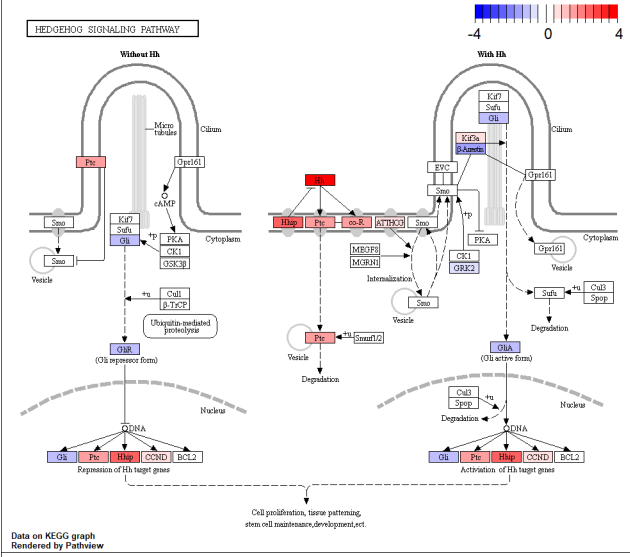

D9

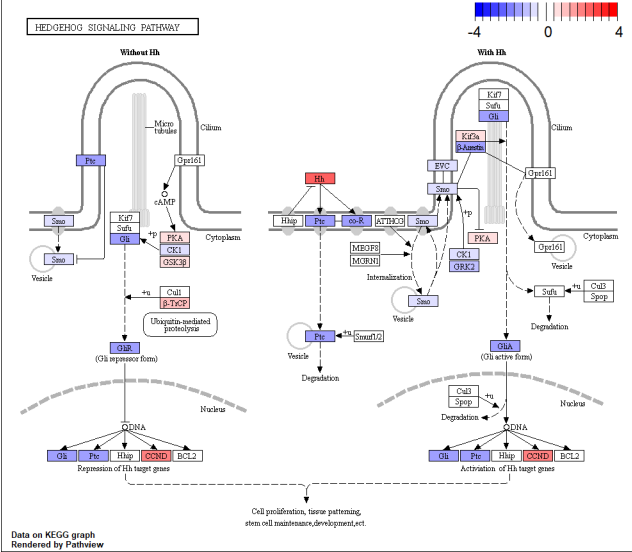

D18

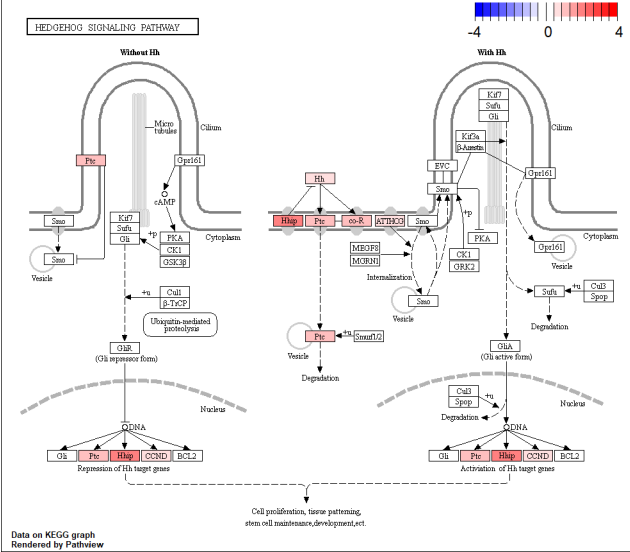

Supplement: Supplementary file 1 [file animals-12-00376-s001.zip › supplementary figures.pdf]
